# The Role of Nanoparticle Elasticity on Biological Hydrogel Penetration

**DOI:** 10.3390/pharmaceutics17060760

**Published:** 2025-06-09

**Authors:** Chathuri I. Sodimanage, Marc Schneider

**Affiliations:** 1 Department of Pharmacy, Biopharmaceutics and Pharmaceutical Technology, Saarland University, Campus C4 1, D-66123 Saarbrücken, Germany; chathuri.sodimanage@uni-saarland.de; 2 PharmaScienceHub (PSH), Campus A2 3, D-66123 Saarbrücken, Germany

**Keywords:** nanoparticle stiffness, deformable particles, biological barriers, hydrogel diffusion, drug delivery, computational modeling, mucus penetration, extracellular matrix permeation, corneal penetration

## Abstract

The latest advancements in nanomedicine have led to increased therapeutic efficacy and reduced complications. However, nanoparticle penetration is significantly influenced by biological hydrogels, such as mucus, the extracellular matrix, biofilms, and nucleoporins. Solely modifying well-studied physicochemical properties like size, charge, and surface chemistry is insufficient to fully elucidate or overcome these barriers. Recent studies have investigated the impact of particle elasticity, a relatively unexplored yet crucial physicochemical property influencing many biological processes. Hence, it is important to explore the impact of particle elasticity on penetrating biological hydrogels. This review examines biological hydrogels’ structural and functional features as diffusion barriers, provides an overview of particle elasticity, key elasticity measurement techniques, and explores strategies for elasticity modulation in nanoparticles, such as composition, crosslinking density, and structural design. Furthermore, nanoparticle penetration mechanisms, influenced by particle deformability, hydrogel mesh size, and adhesive interactions, are investigated by integrating theoretical and experimental findings. The evaluation of experimental data reveals the commonly observed particle elasticity trends in mucus penetration, extracellular matrix permeation, and corneal penetration of nanoparticles. Overall, this review offers valuable insights into designing next-generation nanomedicines capable of overcoming biological barriers.

## 1. Introduction

Nanomedicine has gained significant attention in pharmaceutical research due to its recent advancements in targeted therapy, enhanced drug delivery efficacy, and reduced side effects. Nanoparticles as versatile drug carriers possess several functions such as protecting the active moiety from enzymatic degradation, modulating their pharmacokinetics, and improving drug targeting while reducing the toxicity. However, these performances often result from precise engineering of nanoparticles’ physicochemical properties, including size, charge, and surface modifications, which determine their interactive behavior and in vivo fate in biological systems [1,2]. However, in oral and local administration routes, including nasal, pulmonary, ocular, and intratumoral delivery, nanoparticles frequently encounter biological barriers that can significantly impede drug transport and therapeutic efficacy [3].

Biological hydrogels are a relevant type of biological barriers which protect the tissues and organs by selectively regulating the passage of foreign materials. For topical application at the eye or by inhalation, for example, biological hydrogels serve as robust barriers that prevent the entry of pathogens and foreign substances, while also hindering the diffusion of nanoparticles [4]. Mucus, for instance, entraps the pathogens and particles in the respiratory and gastrointestinal tract [5]. Similarly, extracellular matrix (ECM) controls the interstitial diffusion of particles [6]. In topical drug delivery, not only pericellular hydrogels but also intracellular cytoskeleton and nuclear pore complexes play a distinct role in obstructing penetration [4]. In addition to inherent human biological barriers, bacteria also create a hydrogel barrier known as bacterial biofilms. These biofilms modulate the bacterial environment making them particularly difficult to eradicate [7].

A core challenge that persists in nanomedicine is overcoming these barriers. While conventional physicochemical properties like size, charge, and surface modifications have often been optimized, solely relying on these properties might be insufficient to overcome these barriers. Moreover, the most suitable physicochemical properties facilitating the penetration of one biological barrier may impede or reduce the delivery of nanoparticles at subsequent stages. For instance, although PEGylated nanoparticles exhibit rapid mucus penetration, PEGylation reduces the cellular uptake of the particles [8,9]. Hence, designing a universally competent delivery system is a challenging task.

Recently, nanoparticle elasticity is gaining more and more recognition as a tunable physicochemical property that, alongside size, charge, and surface modifications, contributes to its enhanced biological performances in tumor uptake [10], extended circulation time [11], and protein corona formation [12]. Cevc et al. (2003) [13], one of the very first authors to study particle deformability and penetration of a biological barrier, namely skin, argued that the adaptive structure of elastic particles allows them to fit into the pores of the barrier and penetrate semi-permeable barriers, enabling them to cross it [13,14]. Elasticity-tuned particles also demonstrated enhanced biological hydrogel penetration compared to conventional particles with comparable physicochemical characteristics [15,16].

It is important to understand that both nanoparticles and hydrogel properties play a key role in permeating these barriers. Consequently, understanding the mechanism of penetration into hydrogels is crucial to develop barrier-penetrating drug delivery systems. Despite the advancements in biomedical research, these mechanisms are not fully understood. The complex and highly dynamic properties of biological hydrogels have led to these knowledge gaps. However, a combination of experimental data with computational modeling provides rational explanations for these mechanisms.

For a general description of elasticity effects for nanomedicines, many aspects would also need to be considered such as the circulation time directly impacting on the pharmacokinetics of the carriers and the loaded drugs. This effect will also be balanced with premature drug release which might also be affected by elasticity but there is a lack of data regarding this aspect and thus this aspect could not be included. Similarly, the interaction with cells and the respective uptake is dependent on the protein corona which also depends on elasticity influencing the type and structure of adhered proteins [17,18]. This is a key point for the interaction pattern but the effect of protein corona and elasticity for biological fate is not yet explored and we will therefore not focus on it. Although these functions are individually studied in relation to elasticity, a comprehensive understanding of how nanoparticle elasticity governs the full biological fate of nanocarriers remains to be established.

While recent comprehensive reviews have broadly addressed the effect of nanoparticle elasticity on these biofunctions [19,20,21,22,23], the biological hydrogel barriers present in non-systemic administration routes demand focused attention. These unique hydrogels such as mucus, ECM, and cornea are encountered immediately upon oral, rectal, nasal, ocular, or intratumoral delivery and significantly influence therapeutic outcomes.

This review specifically examines the structural and functional properties of these hydrogels as biological barriers and the interplay between nanoparticle elasticity and hydrogel penetration, aiming to identify the key trends. It is important to understand that both nanoparticle and hydrogel properties play a key role in permeating these barriers. Consequently, understanding the mechanism of penetration into hydrogels is crucial for developing barrier-penetrating drug delivery systems. Despite advancements in biomedical research, these mechanisms are not fully understood due to the complex and dynamic nature of hydrogels. However, a combination of experimental data with computational modeling provides rational explanations for these mechanisms. Hence, we further aim to provide a comprehensive understanding of elasticity-mediated penetration mechanisms by integrating experimental findings with theoretical models.

## 2. Biological Hydrogels as Diffusion Barriers

### 2.1. Overview of Biological Hydrogels

Biological hydrogels are composed of a three-dimensional meshwork of polymers which hold 90–99% of water in their meshwork. The polymer composition can be greatly varied from polysaccharides to complex glycoproteins [4,24]. This network is maintained through molecular entanglements and secondary forces, such as ionic interactions, hydrogen bonding, or hydrophobic interactions [25]. Bio-gels are often composed of biodegradable polymers such as collagen, gelatin, hyaluronic acid, and alginate, which play distinct roles in living organisms [26]. Their functions range from contributing to mechanical support to cells and tissues, tissue repair and regeneration, to lubricating in joints and epithelial surfaces [27,28,29,30].

**Figure 1:**
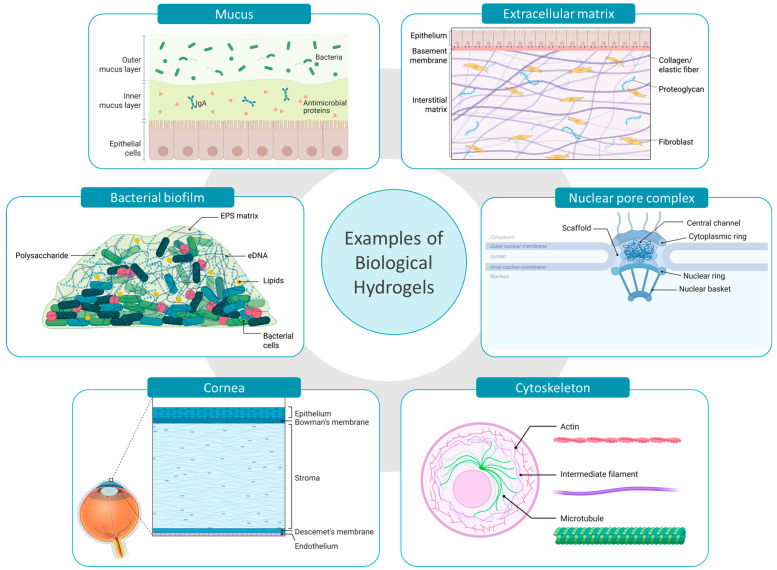
Examples of biological hydrogels acting as bio-barriers in drug delivery. Created with BioRender.com (accessed on 10 May 2025).

More importantly, bio-gels such as mucus, extracellular matrix, and biofilms display a protective role by acting as diffusion barriers [31,32,33]. The viscoelastic matrix created by their hydrated polymer network obstructs the free movement of several particles, pathogens, and chemical entities. This barrier function is not merely physical but also involves dynamic interactions between the penetrating agents and matrix components of the gel. Larger particles are commonly known to be sterically obstructed while charged, hydrophobic or adhesive molecules are subjected to static and dynamic interactions within the gel matrix. This selective permeability regulates the transport of substances to underlying cells or tissues, thereby maintaining physiological balance, preventing infections, and shielding sensitive cells from harmful environmental exposures [24,34]. Biological hydrogels are complex by structure and particle permeation behavior highly depends on the density and structural arrangement of each bio-gel [35]. In the following section, a concise overview of therapeutically important bio-barriers (Figure 1) is discussed.

#### 2.1.1. Mucus

Mucus is primarily located on non-keratinized surfaces, including the respiratory, gastrointestinal, and urogenital tracts. The structural features of mucus vary significantly depending on its location and physiological role, influencing its viscoelastic properties and interactions with pathogens and other substances. Mucus is primarily composed of mucin, a glycoprotein network responsible for the mucoadhesiveness, hydrophobicity, and viscoelasticity of mucus [32]. Other significant constituents present in lower quantities, such as lipids, additional proteins, and DNA, contribute to the matrix’s diffusivity, viscosity and to its defensive barrier against microbes (Figure 1) [36].

#### 2.1.2. Extracellular Matrix

Similar to mucus, the extracellular matrix also possesses a complex hydrogel structure; however, it differs markedly in both its structural constituents and functional roles. ECM provides mechanical support for cells and tissues, functions as a signaling center for cell behavior, and a regulator of cell migration and differentiation [33]. Collagen is the most abundant structural protein present in ECM, providing tensile strength and support to the gel matrix. Working alongside collagen, elastin ensures the low stiffness and high stretchability and storage of elastic energy. The interstitial spaces of the ECM are mainly filled with proteoglycans which retain water, forming a hydrated matrix. In addition, glycoproteins such as fibronectins and laminins are involved in cell adhesion and signaling (Figure 1). However, the architecture of the ECM can greatly vary depending on the specific tissue or organ, with variations in composition and structure [33,37,38].

#### 2.1.3. Cornea

The cornea is the outermost protective layer of the eye, mainly composed of three layers including corneal epithelial layer, stroma, and endothelium. The cornea contains ~78% of water by volume and stroma comprises about 90% of its total thickness. The main structural constituent of stroma is collagen fibrils which are rigidly packed to form lamellae. The proteoglycans present contribute to the hydrogel-like structure, maintaining the spacing between collagen fibrils and the cornea’s hydration. The most predominant cell type in the corneal stroma, the keratocytes, synthesize the stromal ECM components (Figure 1) [39,40]. However, corneal epithelium acts as the main ocular barrier while stroma and endothelium obstruct the transcorneal permeation [41].

#### 2.1.4. Cytoskeleton

When analyzing hydrogel barriers at the cellular level, the cytoskeleton is recognized as a significant barrier involved in regulating endocytosis and intracellular diffusion of nanoparticles within cells [42,43]. The cytoskeleton consists of actin filaments, intermediate filaments, and microtubules, which are arranged as a three-dimensional network to entrap the aqueous cytosol (Figure 1). This protein meshwork is responsible for maintaining the cell shape while organizing internal structures and facilitating intracellular transport [44]. The cytoskeleton forms several compartments inside the cytoplasm where up to 40% of the volume of these compartments is occupied by macromolecules, limiting the diffusion of nanoparticles. Further, the cytoskeleton along with cellular organelles collectively impede the non-directed intracellular motion [42]. It has been reported that the mammalian cell possesses an actin network with pore sizes ranging from 30 to 100 nm to 300–600 nm [45,46]. However, the integrity of the cytoskeleton may be greatly affected by several factors like disease condition, aging, proliferation, and degeneration ultimately influencing the intracellular nanoparticle dynamics [42,47].

#### 2.1.5. Nuclear Pore Complex

The nuclear pore complex (NPC) is an intriguing structure involved in nanoparticulate gene delivery. The NPCs are embedded throughout the nuclear envelope and act as permeability barrier between the nucleus and the cytoplasm [48]. The diffusion barrier of the NPC’s central channel is formed by nucleoporin proteins (NUPs) where the majority of the NUPs possess a repeating unit known as the phenylalanine-glycine (FG) motifs. These FG motifs are highly dynamic and hydrophobic, and self-assembled to form a hydrogel-like structure. The FG domains maintain close contact with the NPC wall constituents, forming a sealed barrier (Figure 1). FG domains strongly restrict the movement of macromolecules while facilitating the passage of nuclear transport receptors (NTRs) and NTR-cargo complexes. However, the mechanism of NTR permeating the solid-like FG barrier is not fully revealed [49,50,51].

#### 2.1.6. Bacterial Biofilms

In addition to human physiological hydrogels, microbial communities also utilize hydrogel matrices for protection and survival. For instance, bacterial biofilms are complex communities of bacteria encased in a self-secreted matrix [52]. Up to 97% of the biofilm matrix is composed of water while the gel-like structure is formed by extracellular polymeric substances (EPS) including polysaccharides, proteins, lipids, and extracellular DNA (eDNA). Polysaccharides are key to adhesion and structural stability, while proteins contribute enzymatic activity and support the matrix structure. Both polysaccharides and proteins protect the bacterial cells, forming a protective environment against other pathogens and antimicrobial agents. Furthermore, the eDNA facilitates horizontal gene transfer between biofilm cells, developing resistance to antibiotics and getting adapted to the environment (Figure 1) [31,53]. However, the bacterial biofilm structure significantly varies based on both the maturity and the specific bacterial species involved [52]. For the treatment of biofilm-based infections the knowledge about those structures and options for overcoming these barriers are essential for future therapeutic success.

### 2.2. Barrier Properties of Biological Hydrogels

The selective diffusion process of biological hydrogels is governed by both size-exclusion and interactive filtering mechanisms. In a gel matrix the polymer entanglements and their crosslinks form a mesh with characteristic pores. However, these pore sizes depend on the polymer chemistry, their concentration, ionic strength, fiber thickness, and cross linker density. In size-dependent permeation, objects smaller than the pore size of the gel matrix might freely pass through the matrix depending on the viscosity of the interstitial fluid while objects exceeding the mesh size are trapped [54]. However, objects with comparable size to the mesh size experience obstruction but penetration is not fully stopped [24,34]. Biological hydrogels present in the human body as well as in animals often display size-dependent filtering. In human endotracheal mucus, 200 nm and 500 nm nanoparticles show diffusion reductions of 5- and 100-fold compared to 100 nm particles [55]. Similarly in porcine intestinal mucus, when the particle size increased from 100 nm to 500 nm, the particle diffusivity was reduced from 2.9- to 20-fold [56]. In the tumor ECM, the diffusion of therapeutic nanoparticles is often obstructed by the increased density and decreased pore size resulting from the rearrangement of ECM components [6]. Nucleoporins present in the NPC possess a mesh size of ~2.5–5 nm and selectively exclude proteins and protein complexes larger than 30–100 kDa, unless they are accompanied by a NTR [57]. Size-dependent filtration is not limited to human and animal tissues. For example, bacterial biofilms, despite having water-filled channels for nutrient and waste exchange, restrict antibiotic penetration based on size [58,59,60].

However, the barrier properties are not solely determined by their mesh size, but also by biochemical and biophysical interactions between the matrix and particles. The human body exhibits several interactive filtering processes. For instance, in the brain’s extracellular space, some large and small molecules display similar microscopic mobility, whereas other molecules with comparable size experience hindered diffusion [4]. The electrostatic interactions between the positively charged particles and negatively charged bio-gel components alter the permeability properties. For example, the negatively charged bacterial exopolysaccharides like alginate or gellan gum significantly control the diffusion of positively charged molecules within biofilms. In a tumor-like ECM model, nanoparticles functionalized with positively charged peptides showed the highest penetration compared to its negative and neutral counterparts [61]. The enhanced penetration may be initially attributed to electrostatic interactions between the positively charged particles and the negatively charged tumor extracellular matrix. These electrostatic interactions are reversible, enabling the particles to attach and detach while moving deeper into the tissues. Furthermore, the concentration gradient created by Donnan interactions helped the particles partition from the surrounding solution into the matrix [61]. Hydrophobic interactions play a role comparable to electrostatic interactions in hindering the penetration and permeation of bio-barriers. Within the mucus barrier, the hydrophobic domains present in mucin entrap hydrophobic particles, including drug delivery systems. These hydrophobic domains of mucus form low-affinity adhesive interactions with the hydrophobic regions of the penetrating object. Although individual interactions are disturbed by thermal energy, a very high number of low affinity interactions are sufficient to immobilize the particles within the matrix [62].

## 3. Understanding Nanoparticle Elasticity

### 3.1. Definition

Elasticity is a relatively unexplored yet key physicochemical property of nanoparticles. It refers to the nanoparticle’s ability to deform under stress and return to its original structure upon the removal of the external force [22]. This property is an intrinsic mechanical property that can be quantified by the elastic modulus. The elastic modulus can be expressed as Young’s modulus, shear modulus, or bulk modulus. When describing nanoparticle’s elasticity, Young’s modulus is the most frequently used, which defines the ratio between tensile stress and tensile strain.

### 3.2. Elasticity Measurement Techniques

**Figure 2:**
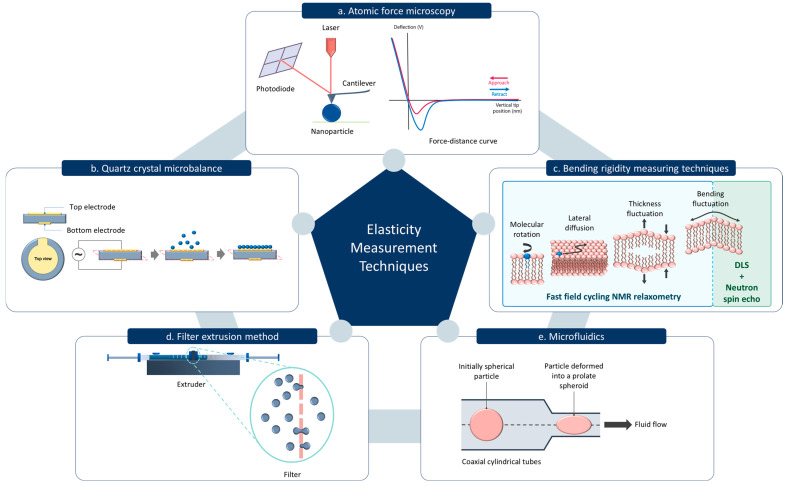
Common techniques for measuring material elasticity. (**a**) Atomic Force Microscopy. (**b**) Quartz Crystal Microbalance. (**c**) Bending rigidity measuring techniques including the fast field cycling Nuclear Magnetic Resonance (NMR) relaxometry and the Neutron spin echo and Dynamic Light Scattering (DLS) measurement. (**d**) Filter extrusion method. (**e**) Microfluidics method.

Nanoparticle elasticity can be experimentally assessed using several methods, with atomic force microscopy (AFM) being the most widely used and the only method that quantifies the stiffness of a single particle [20,21]. AFM consists of a nanoscale tip mounted on a cantilever, which acts as a soft spring. In force spectroscopy measurements, the cantilever tip moves towards the sample until it contacts the sample surface and then bends upwards. This deflection process depends on the properties of the surface and may also be accompanied by a vertical indentation. The cantilever continues to deflect until the applied force reaches a predefined value and then retracts back to its initial position. An optical lever system detects the cantilever’s deflection by projecting a laser beam onto the back of the cantilever and directing it towards a four-quadrant photosensitive detector. Force–distance curves corresponding to the approach and retraction phases are generated by recording the cantilever deflection at each measurement point (Figure 2a). The elasticity of the sample can be determined by analyzing the approach phase of these force–distance curves using several mathematical models [19,63]. Extracting the Young’s modulus from a force–distance curve depends on several factors including the spring constant of the cantilever, compressibility of the sample, and the contact area between the sample and the tip. The contact area depends on the geometry of the tip where spherical, pyramidal, and conical are the most used geometries. Depending on the tip geometry, different formulas of the Hertz mathematical model can be applied to compute the Young’s modulus. However, in practice, this model is applied when indentation depth is less than 10% of the sample thickness [63,64].

Alternative methods of measuring the elasticity of nanoparticles include quartz crystal microbalance (QCM) (Figure 2b) [65]. In QCM, the piezoelectric property of a quartz crystal is utilized to determine the mass change on the surface. When an alternating voltage is applied across the crystal, it oscillates at a specific resonant frequency which is highly sensitive to any mass absorbed onto the crystal surface. The nanoparticles adsorbed onto the QCM sensor introduce a mass load, causing a decrease in the resonance frequency. This frequency shift can be measured to determine the mass of the nanoparticles adsorbed. When QCM is coupled with dissipation monitoring (QCM-D), the energy dissipation of the oscillating crystal can be simultaneously measured with the mass change. Energy dissipation is minimal when a rigid mass is adsorbed. In contrast, if the adsorbed layer is viscoelastic, some of the energy will be lost during each oscillation cycle which can be quantified by the dissipation factor. A highly elastic layer may store higher energy causing a lower dissipation factor. The shear modulus of the particles can be obtained by analyzing the frequency shift and dissipation change as a function of overtone number and surface coverage [66].

The degree of elasticity can be assessed not only by measuring the elastic modulus, but also by calculating a deformability index, which is determined based on the percentage change in particle size under applied stress or deformation conditions [67]. In the filter extrusion method, the deformability index of the nanoparticles is calculated by extruding particles through a filter with 100–200 nm pore size (Figure 2d). Here, the rate of penetration is considered to be directly proportional to the deformability index of the particle [67,68]. Similarly, the microfluidics technique can be utilized to assess the particle deformability under dynamic conditions to quantify, compare, and analyze the characteristics of particles [69]. One study has also validated a microfluidic device to calculate the elastic modulus of microparticles using its kinematics and shape deformation of the particle in an extensional flow (Figure 2e) [70]. Bending rigidity is another important parameter which provides insights into the mechanical properties of lipid-based nanoparticles. The resistance of lipid bilayer membrane to deform or bend is known as the bending rigidity, which is important for nanoparticle mechanical stability and shape [19]. A combination of neutron spin echo techniques with dynamic light scattering measurement can be used to measure the bending rigidity of lipid-based vesicles. Neutron spin echo can be used to study the relaxation dynamics of the membrane undulations, which are related to the bending rigidity (Figure 2c) [71]. In addition, fast field cycling nuclear magnetic resonance relaxometry can be used to evaluate the bending elastic modulus by analyzing the proton spin-lattice relaxation rate, which reflects membrane undulation dynamics. The relaxation rate dispersion of the lipid protons is caused by dynamic processes such as order fluctuations due to shape fluctuations, translational diffusion of the lipid molecules on a curved surface, rotations of the lipid molecules, and fast internal motions within the lipid molecules (Figure 2c) [72,73].

### 3.3. Factors Affecting Nanoparticle Elasticity

The elasticity of nanoparticles is greatly influenced by internal and external factors. Internally, the material properties and structural design, such as degree of crosslinking which changes due to aging [74], play a vital role. Externally, the properties of the surrounding medium such as hydration, concentration of ions, and temperature influence the elastic behavior of the particles [75].

The impact of chemical composition is evident in studies where nanoparticles with comparable size and zeta potential showed different elasticities based on their chemical composition. For instance, solid lipid nanoparticles, polymer/lipid Janus nanoparticles (JNP), and polymer nanoparticles with comparable size and zeta potential showed an elasticity of 0.3 GPa, 0.6 GPa, and 1.1 GPa, respectively, when imaged in air [16]. In hydrogel and gelatin nanoparticles, simply modifying the crosslinking density while maintaining other physicochemical characteristics within comparable limits can significantly change the elasticity. For example, hydrogel nanoparticles exhibit an elasticity ranging from 0.2 kPa to 3 MPa based on their crosslinking [76]. Similarly, gelatin nanoparticles show elasticity values spanning from 2.7 to 14.2 MPa, linked to variations in their crosslinking [77,78,79]. Here, the capacity to interact with water (e.g., swelling) always needs to be considered as it might impact the elasticity values. The elasticity of liposomes is greatly affected by the composition of their lipid bilayer. Varying the phospholipid chain length and saturation allows the tuning of elasticity to a range of 5 MPa to 40 MPa [80]. Liposomes, primarily composed of hydrogenated soybean phospholipids (HSPC) and varying concentrations of cholesterol, showed similar size and morphology, while their elasticity spanned from 11 MPa to 53 MPa [81].

The nanoparticle structural design alterations made by modifying the outer shell and inner core lead to significant elasticity differences. A study on core–shell nanoparticles with PEGylated lipid bilayer and different core compositions such as aqueous, hydrogel, and poly lactic-co-glycolic acid (PLGA) resulted in similar size, shape, and surface chemistry with varying elasticities from 45 kPa to 760 MPa [11]. Similarly, PLGA-lipid NPs with different rigidities were acquired by varying the amount of interfacial water between the PLGA core and the lipid shell. In this study, the Young’s modulus was reported to range from 5 MPa to 110 MPa [15]. Lipid-polymer nanoparticles with consistent sizes exhibited different flexibilities depending on the number of lipid shell layers [82]. Varying the shell thickness of silica nanocapsules can achieve a Young’s modulus ranging from the kPa to MPa range [83].

In addition to chemical and physical modifications, biological modifications can also be used to alter elasticity by biosynthesis methods. This is generally applicable to extracellular vehicles (EVs), where the mechanical properties are based on the type of cell line and how the cell line was manipulated or cultured [84,85,86]. For instance, EVs derived from different types of osteosarcoma cells exhibited a Young’s modulus ranging from 50 to 350 MPa, indicating a considerably high Young’s moduli compared to synthetic liposomes. It has been revealed that membrane proteins present in these vesicles exclusively contribute to their mechanical properties. Exosomes derived from metastatic tumor cells possess an exclusive protein content compared to their nonmetastatic counterparts leading to higher Young’s modulus values [86]. The Young’s modulus of EVs derived from the bone marrow and peripheral blood of clinical hematologic cancer patients showed significant differences compared to those of healthy volunteers [85]. Similarly, highly invasive breast cancer cells secrete small EVs with comparatively lower Young’s modulus than nontumor and less-invasive breast cancer cells [84]. Common mechanical techniques used for EV isolation, such as ultracentrifugation and sonication, also lead to varied elasticities, while some methods cause significant differences in elasticity compared to control EVs. This is mainly due to the potential of these mechanical techniques to damage EV populations through morphological changes and membrane reorganizations, which ultimately affect the fate of EVs [87].

Beyond the intrinsic material properties and structural design, external factors also play a significant role in influencing nanoparticle elasticity. It is well known that the temperature of the material affects its elasticity. As the temperature changes, it affects the amplitude of the atomic vibrations leading to stretching or compressing of the atomic bonds, ultimately affecting its elasticity [88]. Additionally, the medium in which nanoparticles are dispersed is important for measuring their elasticity. If the particles, especially polymer and hydrogel nanoparticles, are dispersed in an aqueous medium, water molecules are absorbed into the nanoparticles, acting as plasticizers. These absorbed water molecules increase the free volume within the polymer structure and enhance the mobility of the polymer chains, leading to a transition from a rigid, glassy state to a softer, more rubbery state compared to their dried state in air [21,89].

## 4. Nanoparticle Elasticity and Penetration Mechanisms in Biological Hydrogels

Nanoparticle penetration through biological hydrogels is a complex process influenced by the properties of both nanoparticles and the hydrogel matrix. Biological hydrogels serve as selective barriers, employing size-based exclusion and interactive filtering as previously discussed [34]. Meanwhile, many studies have shown that the commonly known properties of nanoparticles, including size, shape, surface charge, and composition, play a crucial role in penetrating these barriers [90,91,92,93,94,95]. Another key parameter influencing penetration, common to both nanoparticles and the hydrogel network, is their elasticity.

### 4.1. The Effect of Nanoparticle Elasticity

In recent years, drug delivery research has focused on the impact of nanoparticle elasticity on their behavior in biological systems [10,11,77]. The initial findings on the effect of particle deformability in pharmaceutical applications were demonstrated with “skin-penetrating” liposomes. Cevc et al. (2003) [13] explained that a driving force, sufficient to drag liposomes larger than skin openings, was required for successful penetration. They claimed hydration–gradient–driven transport as the driving force. Furthermore, they explained that the energy needed for an intact vesicle to enter a pore depends on the size and elasticity of the vesicle membrane, with low vesicle rigidity favoring energetically inexpensive membrane deformation. Consequently, ultra-flexible liposomes were shown to penetrate successfully, whereas conventional, less flexible liposomes failed [13,96]. Similar behavior of deformable particles was also observed with hydrogel penetration where the deformable particles alter into an ellipsoidal shape to pass through the polymeric network of hydrogels while maintaining structural integrity [97]. This deformability contrasts with rigid nanoparticles which are unable to adapt their conformation and may experience steric obstruction and reduced penetration [2,60,61]. Alternatively, excessively soft nanoparticles tend to adhere to the polymeric fibers, thereby decreasing the diffusivity [15].

### 4.2. The Effect of Hydrogel Mesh Size and Polymer Network Elasticity

The degree of crosslinking and mesh size have a direct impact on nanoparticle mobility. The ratio between the nanoparticle diameter and hydrogel mesh size is defined as the confinement ratio, which determines whether nanoparticles move freely or encounter obstruction within the hydrogel. A high confinement ratio results in a mesh size smaller than particle size, restricting particle movement and leading to localized or subdiffusive behavior [98,99]. However, the dynamic nature of the polymer chains may exhibit varying degrees of flexibility and rearrangements within the hydrogel. Highly flexible polymer chains may encounter transient expansions, facilitating the mobility pathway of the particles, while a stiff matrix creates a barrier [100,101].

### 4.3. Computational Modeling of Penetration Mechanisms

Investigating the exact nanoparticle penetration mechanisms and individual affecting parameters within hydrogels experimentally is a highly complex task. Therefore, theoretical and computational models serve as effective alternatives for exploring this phenomenon. Yu et al. (2022) [102] propose a key model focusing on three parameters affecting particle penetration: stiffness of the nanoparticle, mesh size, and affinity strength of the polymer network. The Mean First Passage Time (MFPT) theory, which calculates the time for a nanoparticle to travel one lattice length of the gel, considers only the nanoparticle and hydrogel properties, which can only apply to stiff nanoparticles. Instead, the study conducted by Yu et al. (2022) [102] steps further, considering the internal energy changes in the particle during deformations. It demonstrates that, when the affinity strength is less, adhesive energy will be lower than the deformation energy of the particle and the initial spherical shape of the particle will not be disturbed while it experiences a high energy barrier. Similarly, when the affinity strength and adhesive energy exceed the deformation energy, an energy barrier is created once again [102]. Concurrently, high adhesive energy increases both the particle deformation and the contact area between the particle and the polymer network [102,103]. However, with optimal affinity strengths, adhesive energy will be counterbalanced with deformation energy, resulting in a low energy barrier and increased diffusion rate. Hence, the highest diffusion rates were observed with semi-elastic particles, further validating the experimental findings discussed later in this review. In conclusion, to achieve a high diffusion rate, adhesive energy must increase proportionally with particle stiffness to counterbalance the deformation energy [102].

**Figure 3:**
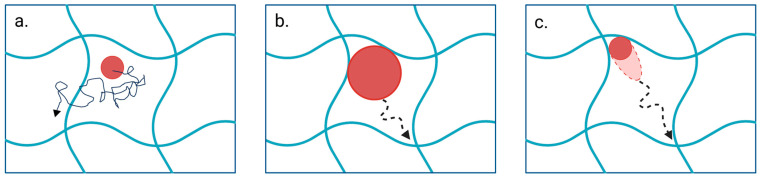
Influence of particle properties and hydrogel network characteristics on diffusion mechanisms. (**a**) Soft particles randomly diffuse through hydrogels with large mesh size and weak affinity strengths. (**b**) Rigid particles diffuse in smaller mesh-sized, stronger affinity hydrogels. (**c**) Semi-elastic particles diffuse in large mesh-sized, strong affinity hydrogels by deforming under attraction forces. Created with BioRender.com (accessed on 25 April 2025).

Hopping behavior is one of the diffusion mechanisms observed in nanoparticles, in which the particles hop between neighboring polymer entanglements [102,104]. Yu et al. (2022) [102] explained that increased mesh size reduces the adhesive interactions between the particles and other attraction points of the mesh, resulting in a tight attachment of particles to one region [102]. When the affinity of the hydrogel increases, Tian et al. (2019) [103] also explained a similar tight attachment of particles to the hydrogel [103]. Therefore, regardless of the particle stiffness, a small mesh size promotes the hopping diffusion of the particle. With optimal mesh size, diffusivity is affected by the stiffness of the particle and adhesion strength. Semi-elastic particles experience attractions from different adhesive regions while deforming themselves and possessing a high diffusion rate [102]. A simulation study conducted on a series of liposomes with different elasticities demonstrates a similar concept, where semi-soft liposomes exhibit an attraction toward one corner of the network and then deform into an ellipsoidal shape due to the attraction to another corner. Due to these attraction forces and deformation energy, liposomes diffuse from one corner to the other. Further, this study has explored the effect of liposome phase transition temperature (T_m_) on particle penetration. Simulated data revealed that at low temperatures, particle diffusivity is a minimum, and upon temperature increment, the semi-soft particles showed a diffusivity of ~1.8- and 2.7-fold higher than the hard and the soft liposomes, respectively. Further increase in temperatures led the hard particles to diffuse ~1.7- and 3.3-fold higher than semi-soft and soft liposomes, respectively. This depicts the effect of temperature on elasticity, which ultimately affects the penetration efficacy [97]. Concluding these results, Yu et al. (2022) [102] suggested that soft particles are better applicable in penetrating hydrogels with large mesh size and weak affinity strengths (Figure 3a), while rigid particles are more prone to diffuse in smaller mesh-sized, stronger affinity hydrogels (Figure 3b). Semi-elastic particles are more suited to diffuse in large mesh-sized, strong affinity hydrogels (Figure 3c) [102].

## 5. Experimental Studies on the Effect of Nanoparticle Elasticity on Hydrogel Penetration

Experimental studies reveal valuable insights into the effects of elasticity in vitro and in vivo setups. Among all the biological hydrogels present in the body, the impact of particle elasticity on penetration was often studied in mucus [15,16,97,105,106,107]. Very few studies have investigated permeating tumor matrix [15,80,108] and penetrating the ocular barrier with deformable particles [68,109,110,111].

### 5.1. Penetration Across the Mucus Barrier

Mucus is a highly dynamic hydrogel where the crosslinked polymer network of mucin acts as a selective barrier due to its sticky mesh structure and interaction with other mucosal components [112]. In drug delivery, penetrating this barrier may require manipulating the physicochemical properties of the nanoparticles, including their elasticity. A recent study on polymer/lipid JNP for nose-to-brain drug delivery has compared three types of nanoparticles with comparable sizes and charges but different stiffness on penetrating healthy simulated nasal mucus. Elasticity measurements of the particles were performed in air and values were in the GPa range. JNP reported a Young’s modulus (0.693 GPa) 2-fold higher than solid lipid nanoparticles (0.362 GPa) and ~1.5-fold lower than polycaprolactone particles (1.105 GPa). The fact that all samples were measured in air likely explains these remarkably high Young’s moduli. Here, the anisotropic geometry and semi-elastic property of JNP contributed to the highest diffusion rate, reaching the deepest segments of the mucus barrier [16]. In another study comparing liposomes and lipid/polymer hybrid nanoparticles, the particles were modified with Fc receptor ligand (FcBP) at a similar rate to target the bronchial mucosa. The liposomes (soft particles) and hybrid nanoparticles (stiff particles) indicated a shear modulus of 84 kPa and 2020 kPa, respectively. Soft nanoparticles showed a higher sputum penetration efficiency in cystic fibrosis sputum. However, the anti-inflammatory study on acute lung inflammation rat model revealed that pulmonary retention and therapeutic efficacy were greater with stiff nanoparticles than their soft counterpart. Further, the investigation of the mechanism demonstrated that FcBP ligands are better expressed on stiff particles, enhancing the targeting efficiency. The endocytosis and exocytosis of the bronchial epithelium were also enhanced due to stronger engagement of stiff particles with actin filaments and triggered Ca^2+^ signal [65].

Some lipid-based nanoparticles with intermediate stiffness achieve the optimal intestinal mucus penetration by balancing deformability and structural integrity [15,97,107]. Yu et al. (2018) [15] investigated the impact of polymer/lipid hybrid nanoparticle elasticity on intestinal mucus penetration. The semi-elastic particles’ Young’s Modulus (50 MPa) was 10-fold higher than soft particles (5 MPa) and 2-fold less than the rigid particles (110 MPa) and reported the highest penetration efficiency [15]. Similarly, a study on the penetration of lipid nanovesicles in rat intestinal mucus investigated the effect of T_m_ and elasticity on penetration efficiency. Here the hard, semi-soft and soft particles reported a Young’s modulus of ~28 MPa, ~15 MPa, and ~1 MPa, respectively. This study revealed that the superior liposome penetration was achieved when the temperature was set around T_m_ of the particle. At 37 °C, semi-soft liposomes showed the highest diffusivity with a mean-squared displacement (MSD) ~13.4- and 3.5-fold higher than that of soft and hard particles, respectively [97]. Following the same trend, FcBP ligand modified PLGA-lipid nanoparticles with different elasticities (Young’s modulus of stiff, semi-elastic, and soft particles = 2.118 MPa, 712 kPa, and 85 kPa, respectively) showed that the highest porcine mucus penetration efficiency and fastest diffusion is by semi-elastic particles regardless of ligand modification or not. However, consistent with prior discussion, targeting efficiency was higher in stiff particles as the ligand receptor interactions are facilitated by the rigid surface of the particle [107]. An opposing trend was reported in an in vivo mucus penetration study, where insulin-loaded self-emulsifying lipid nanoparticles (Young’s modulus of hard, medium-hard, and soft particles = 111 MPa, 55 MPa, and 15 MPa, respectively) with the highest stiffness showed rapid and greater permeation in rat intestinal mucus [105]. In a similar direction, zwitterionic hydrogel nanoparticles showed the highest intestinal absorption with rigid particles (Young’s modulus = 165.2 MPa) which were approximately 2-fold and 37-fold stiffer than medium-hard (Young’s modulus ≈ 70 MPa) and soft particles (Young’s modulus ≈ 5 MPa), respectively. However, the highest porcine mucus penetration efficiency was shown by soft particles with MSD 2.9 times higher than that of rigid particles [106].

In conclusion deformable particles showed the highest penetration both in intestinal and nasal mucus while stiffer particles can be better for targeting and sometimes even penetration depending on the specific mucus and biological context. The ideal nanoparticle elasticity for drug delivery is highly dependent on the application.

### 5.2. Extracellular Matrix Permeation

Permeating ECM is crucial in various biological and medical applications, particularly in cancer therapy and tissue engineering. However, the complex network of biomolecules in ECM possesses an irregular pore morphology, forming a barrier to the drug delivery systems [35]. A study conducted on the impact of the mechanical properties of lipid vesicles on their BxPC-3-HPSC tumor uptake has specifically investigated the role of liposome rigidity on tumor ECM diffusion. The mechanical properties of liposomes were modified by varying the chain length and saturation of the acyl chains of the phospholipids. Liposome rigidity significantly increased with the length of saturated acyl chains, while the introduction of a single double bond caused a drastic decrease in rigidity, regardless of chain length. Out of soft (5.8 MPa), semi-elastic (19.9 MPa), and stiff (42.8 MPa) liposome formulations, semi-elastic liposomes showed a significantly higher MSD in the simulated tumor ECM compared to its soft and stiff counterparts [80]. Similarly, Yu et al. (2018) [15] conducted another study on elasticity-modified particle penetration in the same type of tumor. Following peritumoral injection of soft (5 MPa), semi-elastic (50 MPa), and rigid (110 MPa) particles into tumor-bearing mice, they found that only the semi-elastic particles showed efficient internalization into both the tumor tissue and cancer cells. The semi-elastic particles distributed throughout the tumor interstitial matrix, whereas the other particles were only detected in perivascular regions [15]. When ovarian cancer-bearing mice were treated via tail-vein injection with formulations of stiff (24 ± 14 kPa) and soft (6 ± 3 kPa) layer-by-layer nanoparticles, soft particles showed twice the tumor accumulation compared to the stiff particles [108]. These findings prove the significance of particle flexibility to permeate through ECM by transient shape alterations. Nevertheless, it is crucial to acknowledge that ECM structure and density vary depending on its physiological function. Hence, the permeation behavior of the particle varies according to the type of ECM. For instance, both exosome-like vesicles and liposomes showed higher effective diffusion coefficients in less dense muscle ECM than more dense cartilage and tunica albuginea ECMs [35]. Accordingly, it is important to optimize the particle elasticity to permeate the intended type of ECM.

### 5.3. Penetration Across the Cornea

In ocular drug delivery, the most compliant route of administration is topical, which is often impeded by the corneal barrier (ocular bioavailability < 5%). Over the years, nanomedicine has depicted promising results in ocular drug delivery, particularly with lipid-based particulate systems. It has also revealed that deformable nanoparticles outperform conventional particles in overcoming the corneal barrier with respect to the delivered drug amount [68,109,110,111].

A study aimed to deliver coumarin-6 (cou-6) by corneal permeation has compared the corneal penetration ability in vivo with dipotassium glycyrrhizinate modified deformable nanoliposomes and conventional liposomes (CL). Here, the elasticity of the particles was evaluated by the extrusion method, where both particles of around 100 nm size were extruded through a 20 nm pore-size filter. Only deformable particles were capable of penetrating with a size modification to 65 nm. The cou-6 concentrations in both mouse and rat corneas were reported to be higher for deformable particles compared to conventional particles. However, both particles were accumulated in the corneal epithelium, failing to penetrate the deep corneal tissue [111]. A study comparing ultra-deformable bilosomes (UBs), conventional bilosomes, and niosomes on ex vivo corneal permeation considered the percentage of particle size variation after extrusion as a parameter to compare the elasticities. UBs exhibited the highest deformability and permeation in albino rabbit cornea, while conventional bilosomes and niosomes showed the second and third highest deformability and permeation, respectively [110]. The spanlastic (SVs), a surfactant-based vesicular system, demonstrated a 67% improvement of corneal permeability in ex vivo porcine cornea compared to niosomes formulation. In this study, elasticity was also assessed by the extrusion method, where SVs exhibited the least percentage of size variation compared to niosomes, indicating superior elasticity, which in turn contributed to enhanced corneal permeation [109]. Chen et al. 2016 [68] calculated the deformability index as a parameter of elasticity using the extrusion method. Here, deformable liposomes (DL) and CL exhibited a deformability index of 12.08 and 4.67, respectively. Enhanced rabbit corneal permeation was exhibited by DL, where permeation was further enhanced when the particles were coated with chitosan [68].

One key aspect highlighted by these studies is that the more deformable particles are more prone to penetrate the cornea of rabbits and mice, reinforcing the concept that elastic particles squeeze through the transient pores of the cornea. However, it is difficult to compare or set limits for ideal deformability since most of the studies used indirect methods like extrusion to evaluate elasticity. In addition, the penetrated amount is only described in relation to another system but cannot be compared on a quantitative base, which makes a comparison between different works nearly impossible.

## 6. Discussion

This review outlines the current understanding regarding the influence of nanoparticle elasticity on the penetration into various types of biological hydrogels. The compiled data indicate that optimizing nanoparticle mechanical properties offers a promising strategy for overcoming formidable biological barriers such as mucus, cornea, and the extracellular matrix. However, the optimal elasticity values detailed in Table 1 suggest that establishing universally optimal elasticity values remains inconclusive.

A major challenge hindering definitive conclusions and cross-study comparisons is the lack of standardization in elasticity measurement and reporting. Techniques like AFM, QCM-D, and filter extrusion use different aspects of mechanical responses such as Young’s modulus, shear modulus, and deformability index to define the elasticity or deformability of the particle. Even though the same technique was employed, elasticity values may vary depending on the experimental setup. For instance, in AFM, Young’s modulus values are significantly influenced by measurement conditions such as temperature, humidity, vibrations, and system calibrations, as well as sample-related factors like hydration state and preparation methods, making it difficult to compare the absolute values [21,113]. Additionally, the particle properties like material compositions, surface chemistry, and experimental condition, such as in vitro or in vivo, might play a crucial role in elasticity-related particle behavior. More importantly, highly dynamic hydrogels, such as intestinal mucus, do not maintain a universal composition across all the experimental setups. Instead, it may vary according to the host microbiota, nutrition, intestinal motility, and secretions, leading to structural and functional differences and varied nanoparticle behavior within [114].

**Figure 4:**
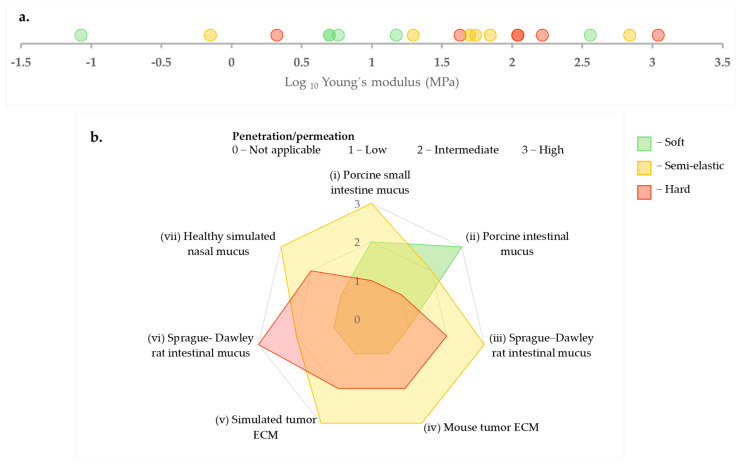
(**a**) Comparison of Young’s modulus values (MPa, log scale) as measured by AFM with corresponding particle stiffness classifications (soft, semi-elastic, hard) as presented in the literature [15,16,80,105,106,107]. (**b**) Illustration of the penetration and permeation patterns exhibited by the aforementioned particles in different hydrogels (i) [107], (ii) [106], (iii) and (iv) [15], (v) [80], (vi) [105], (vii) [16].

A recurring trend, observed across different types of hydrogels such as mucus (nasal, intestinal) and ECM, is the enhanced penetration of intermediate or semi-elastic nanoparticles [15,16,80,107]. Furthermore, computational models supported this idea, indicating that semi-elastic particles can optimally balance deformation energy and adhesive interactions within the polymer network under specific conditions of mesh size and affinity [97,102]. However, drawing a universal conclusion is more complex. Firstly, classification of particles as soft, semi-elastic, and hard is study-specific and depends on the relative comparisons of particles within the study. For instance, Yu et al. (2022) [107] define particles with a Young’s modulus of 2 MPa as hard, whereas Yu et al. (2018) [15] consider 5 MPa as soft, which is more than twice the value used in the former study to define completely different classes of particles [15,107]. Figure 4a presents elasticity values from six different studies, all measured using AFM, along with their respective classifications. The figure clearly illustrates the wide range of these Young’s modulus values, spanning from kPa to GPa, and how each study categorizes them in relation to the particles they investigated. Next, Figure 4b depicts their penetration behaviors across various hydrogel types. This highlights that drawing conclusions focusing solely on stiffness classification can be misleading when the absolute Young’s modulus values are not considered.

However, careful observation of elasticity study data reveals that tuning the elasticity of particles plays a crucial role in successfully penetrating highly complex biological hydrogels. Particles must retain sufficient structural integrity to navigate the hydrogel mesh while possessing enough deformability to squeeze through, balancing propulsion against steric hindrance and mucoadhesion. Yet, considerable research is still needed in both research setup and validation studies to establish elasticity as a clinically important physicochemical parameter.

## 7. Conclusions and Future Directions

Nanoparticle elasticity demonstrates equal importance to other physicochemical properties like particle size, charge, and surface chemistry in drug delivery research. This review highlights a relatively unexplored approach for overcoming biological barriers as an interplay between the mechanical properties of the nanoparticles and the hydrogel structural dynamics. Particles maintaining structural integrity while deforming within optimal limits demonstrate superior performance in penetrating biological hydrogels. Theoretical modeling data supported with experimental findings provides valuable insights to understand the underlying mechanisms of nanoparticle penetration in hydrogels.

However, several challenges remain unaddressed, including the complications in cross-study comparisons due to the lack of standardization. For example, classifications of particles as soft, semi-elastic, or rigid depend on the limits set by each study. Here a quantifiable parameter would be necessary allowing to compare the performance over elasticity and not just relative to other systems used by the authors. It is important to understand that the elasticity value, whether expressed as Young’s modulus or diffusivity index, does not represent a single material property, but rather reflects the combined influence of constituent materials and their structural arrangements within the nanoparticles. Hence, variations in both measurement techniques (e.g., AFM, QCM, extrusion) and experimental conditions further complicate the establishment of universal elasticity thresholds. Additionally, biological hydrogels exhibit dynamic and heterogeneous compositions, leading to study-specific particle behaviors within the hydrogel. Current research is only limited to a few bio-gels like mucus, leaving significant opportunities to explore more on other barriers such as intracellular cytoskeleton, nuclear pore complexes, and extrinsic biological systems such as bacterial biofilms which present equal importance in biomedical research. However, the vast differences in the protocols applying the particles to the biological systems also complicate the comparability and does not allow for a clear conclusion on the effect of particles’ elasticity yet. As a consequence, the picture does not look so clear as deformability and pore size will be the only driving forces, which is well supported by first modeling approaches.

In summary, while current investigations provide valuable insights into the effect of particle elastic behavior on hydrogel penetration, key areas of interest remain for future exploration. The scope for future studies expands from investigating a broader spectrum of biological hydrogels, standardizing protocols for elasticity measurement, conducting comparative studies on measurement techniques, to integrating machine learning to predict optimal elasticity for specific hydrogel barriers. These advancements in bio-barrier penetration studies may lead to the next-generation nanomedicines which can be precisely engineered to overcome biological barriers, enhancing therapeutic efficacy.

## Figures and Tables

**Table 1:** Nanoparticle deformability trends and their penetration behavior in biological hydrogels. Deformability was measured by QCM, AFM and filter extrusion method.

	Type of Nanoparticle	Deformability	Type of Barrier & Penetration/Permeation Behavior	Ref.
a.	Liposomes with/without PLGA core	Soft—84 kPa Stiff—2020 kPa	Cystic fibrosis sputum Soft particles—higher sputum penetration efficiency	[65]
b.	Layer-by-layer Nanoparticles	Soft—6 kPa Stiff—24 kPa	Mouse tumor ECM Soft particles—highest tumor accumulation	[108]
c.	JNP Solid lipid nanoparticles Polymer nanoparticles	Soft—362 MPa Semi-elastic—693 MPa Hard—1105 MPa	Healthy simulated nasal mucus Semi-elastic particles—highest diffusion rate	[16]
d.	Liposomes with/without PLGA core	Soft—85 kPa Semi-elastic—712 kPa Stiff—2118 kPa	Porcine small intestine mucus Semi-elastic particles—highest penetration efficiency and fastest diffusion Stiff particles—highest targeting	[107]
e.	Self-nanoemulsifying drug delivery system	Soft—15 MPa Medium-hard–55 MPa Hard—111 MPa	Sprague–Dawley rat intestinal mucus Hard nanoparticles—rapid and greater permeation	[105]
f.	Zwitterionic hydrogel Nanoparticles	Soft—~5 MPa Medium-hard—~70 MPa Hard—165.2 MPa	Porcine intestinal mucus Soft particles—highest mucus penetration efficiency Hard particles—highest intestinal absorption	[106]
g.	Chain lengths and saturation modified liposomes	Soft—5.8 MPa Semi-elastic—19.9 MPa Hard—42.8 MPa	Simulated tumor ECM Semi-elastic particles—highest tumor ECM diffusion	[80]
h.	Liposomes with/without PLGA core	Soft—5 MPa Semi-elastic—50 MPa Hard—110 MPa	Sprague−Dawley rat intestinal mucus Semi-elastic particles—highest penetration efficiency Mouse tumor ECM Semi-elastic particles—highest tumor interstitial matrix distribution	[15]
i.	Liposomes	DL—size reduction after extrusion—65 nm CL—extrusion was not possible	Mouse cornea and rabbit cornea DL—highest cou-6 accumulation in both mouse and rabbit cornea	[111]
j.	Bilosomes vs Niosomes	Size change after extrusion UBs—21.8% Conventional bilosomes—28.9% Niosomes—39.9%	Albino rabbit cornea UBs—highest permeation	[110]
k.	Spanlastics vs. niosomes	Size change after extrusion Spanlastics—12.0% Niosomes—26.2%	Porcine cornea Spanlastics—highest permeability	[109]
l.	Chitosan-coated liposomes	Deformability index of DL—12.08 CL—4.67	Albino rabbit cornea DL—highest corneal permeation	[68]

## Data Availability

This article does not report any original data, and the research described is based on a review of existing literature.
